# Capillary Electrophoresis and Atomic Absorption Spectrometry—A Rare Yet Valuable Liaison, Also from a Pharmaceutical Perspective

**DOI:** 10.3390/molecules31132209

**Published:** 2026-06-23

**Authors:** Daniel Baecker

**Affiliations:** Department of Pharmaceutical and Medicinal Chemistry, Institute of Pharmacy, Freie Universität Berlin, Königin-Luise-Straße 2+4, 14195 Berlin, Germany; d.baecker@fu-berlin.de

**Keywords:** atomic absorption spectrometry (AAS), capillary electrophoresis (CE), ET AAS, human serum albumin (HSA), hyphenation, interface, metal analysis, pharmaceutical analysis

## Abstract

Both capillary electrophoresis (CE) and atomic absorption spectrometry (AAS) are analytical techniques of high performance given their efficient separation and their sensitive detection, respectively. They are predominantly adduced as standalone techniques, also widely used in pharmaceutical sciences. However, with CE, only indirect detection or direct detection of metal complexes using additives to the background electrolyte is feasible, while AAS is not capable of separating different compounds of the same metal. Combining these two techniques would compensate for these limitations by complementing each other. Surprisingly, the hyphenation of CE and AAS represents rather a rare liaison. Therefore, the aim of the current perspective was to shed light on examples where the hybrid technique of CE-AAS was successfully applied thus far. In this context, particular attention was paid to the extent to which the studies and the analytes under investigation might also be relevant from a pharmaceutical point of view. It revealed that the combination of CE and AAS has great potential for research in pharmaceutical sciences. With respect to online hyphenation, this would be even better if interfaces were more widely available. Nevertheless, this overview demonstrates that CE-AAS is a valuable combination. Its potential just needs to be exploited.

## 1. Introduction

Metal analysis plays a significant role in life sciences. The interdisciplinary research field referred to as “metallomics” generally focuses on metal-containing compounds and their interactions with biomolecules [1]. This is relevant, for example, when it comes to the effects of drugs. In the pharmaceutical sciences, studying metals has an important function with regard to the safety and quality of drugs, the optimization of the production process, or the development of new drug candidates.

In particular, traces of metals may result from the production process (e.g., abrasion from the production equipment or contamination of raw materials) and can affect the quality of drugs [2]. Rigorous limits must be adhered to, especially in the case of toxic heavy metals. In biopharmaceuticals, i.e., drugs generated with biotechnological approaches, different concentrations of metals can influence the growth of microorganisms and, consequently, the yield of the produced drug [3]. In addition, there is extensive research on metal-based compounds as novel drug candidates, such as those with antibacterial [4] or anticancer activity [5].

All of these issues require analytical methods for the separation and quantification of metal-containing agents. Given the charged nature of such species in biological systems, capillary electrophoresis (CE) reveals a suitable separation method for inorganics [6]. It is used to separate bioactive metal complexes and metabolites thereof. By investigating the release of the metal component from the intact complex, conclusions can be drawn about its stability. In addition, interactions with biomolecules, which serve as targets for such drugs, can also be studied with CE [7,8,9]. Further applications of CE in pharmaceutical analysis have been reviewed before [10,11]. Despite this wide range of applications, CE comes along with a few limitations, such as restricted detection in the case of samples with low concentrations [12]. Therefore, sensitive techniques for quantification are required.

One established method for the quantification of metals is atomic absorption spectrometry (AAS) [13]. In the field of pharmaceutical sciences, AAS is widely used in the study of medicinal plants [14], antitumor compounds [15], and in antibacterial research [16]. In addition, it is applied to determine trace elements in pharmaceutical preparations [17] or for the purpose of cleaning validation in the pharmaceutical industry [18].

Both CE and AAS offer advantages. CE is known for its competitive level of resolution, inexpensiveness, short analysis times, and small sample volumes in the single-digit or low double-digit nanoliter range [19]. The latter are particularly useful when analyzing biological samples. Moreover, CE is easy to use and allows for simple automation [20]. AAS is characterized by very high sensitivity, high selectivity (based on the metal-characteristic wavelength) [21], rapid analysis (e.g., compared to high-performance liquid chromatography [22]), and also requires small volumes of analyte [23]. However, depending on the sub-technique of AAS, sample volumes in the low double digit microliter range for electrothermal AAS (ET AAS) or in the range of about 1 mL for flame AAS (F AAS) are required. Thus, the sample volumes in AAS are higher than in CE.

Although the two methods are widely used independently for drug-related applications, hyphenating CE as a separation technique of high resolving performance with AAS as a very sensitive detector would represent a powerful tool to analyze biological samples within the life sciences in general, or for pharmaceutical approaches in particular. However, AAS is not capable of separating species originating from the same metal [24], whereas CE is hampered by its inability to detect compounds directly without additives to the background electrolyte [12]. In these regards, the two techniques can complement each other perfectly. A review of the literature revealed that the techniques CE and AAS are rarely combined. This provides the motivation for the current perspective, namely to present examples of the hybrid use of CE and AAS and thus to bring the liaison of these two methods to the attention of analysts, hopefully also to exploit it in pharmaceutical applications.

## 2. Examples of Applications of CE Hyphenated with AAS

### 2.1. Examples of Offline Hyphenation of CE-AAS

The trace element chromium is of pharmaceutical interest, for example as trivalent chromium ions are constituents of the glucose tolerance factor [25]. Therefore, deficiency of trivalent chromium might contribute to the development of insulin resistance in patients with diabetes [26]. Due to its importance for the proper performance of the human organism, chromium is contained in multimineral pharmaceuticals intended for patients suffering a deficiency of this trace mineral [27]. This context explains why the quantification of trivalent chromium, assessed by AAS, plays a role in pharmaceutical and biomedical analysis [28]. In addition, monitoring of chromium is important given the carcinogenicity of hexavalent chromium [29].

As analysis of chromium by AAS is based on the atomic state of the metal, no distinction between chromium species of different oxidation states can be made using this technique [30]. Therefore, a technique to separate trivalent and hexavalent chromium is required. This can be achieved by CE, as trivalent chromium mainly occurs as a cation (Cr^3+^), while hexavalent chromium exists in an anionic manner (Cr_2_O_7_^2−^). Given the different charges and electrophoretic mobilities, the cation and the anion are thought to migrate to the cathode and anode, respectively. Considering the electroosmotic flow, the separation of dissolved ions (i.e., electrolytes, both cations and anions) proceeds towards the cathode. Cations elute first, while the moving of neutral species is slower, finally followed by anions [31].

The approach of combining CE and AAS for the speciation of chromium was followed by Kuldvee et al. [24]. In this particular case, the hyphenation of both techniques was offline. This means that a preparative separation and collection with CE occurred first, followed by the element-specific detection using ET AAS without direct constructional connection of both devices. Since capillaries made of Teflon with a wider inner diameter (i.e., 300 µm) compared to conventional capillaries (inner diameter 50–70 µm) were used, both injection of larger sample volumes and collection of the separated fractions were feasible. The CE procedure was appropriate to separate trivalent and hexavalent chromium in aqueous samples. The collected fractions were directly used for quantification without further mineralization. The atomization of chromium during the ET AAS analysis was carried out at 2600 °C. A limit of detection of 1 ng mL^−1^ for both trivalent and hexavalent chromium was achieved, which could be further optimized according to the authors’ suggestion.

**Figure 1:**
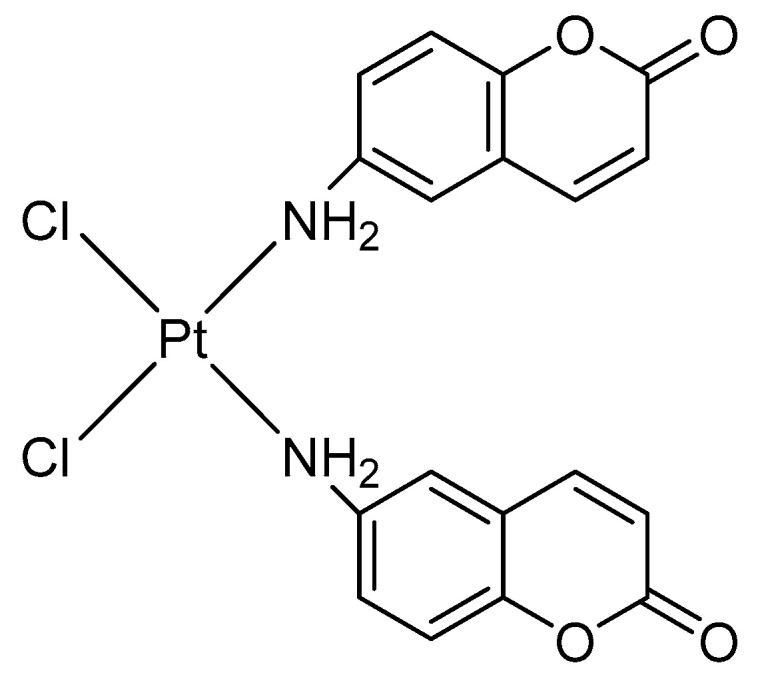
Chemical structure of the antitumor platinum(II) complex, whose binding to DNA was determined using offline hyphenation of CE with GF AAS.

Another study employing the offline coupling of preparative CE with graphite furnace AAS (GF AAS) was reported by Deforce et al. [32]. The research work deals with the analysis of complexes formed by calf thymus deoxyribonucleic acid (DNA) with a platinum(II) complex. The latter contains 6-aminocoumarin ligands (see Figure 1) and proved to exhibit anticancer activity [33].

In general, platinum complexes have a special relevance both in clinical use [34] as well as in pharmaceutical-medicinal research to develop novel anticancer drugs [35]. Platinum complexes predominantly exert their biological activity by inducing apoptosis as a result of binding to the DNA [36]. Therefore, investigating the binding behavior to DNA is of utmost interest when designing new platinum-based drug candidates. In the case of studying the 6-aminocoumarin-bearing platinum(II) complex, incubation of calf thymus DNA was carried out, followed by enzymatic hydrolysis (DNAse I and nuclease P1), yielding a hydrolysate of platinated DNA. The latter was subject to CE analysis. The fractions obtained in this procedure were collected using a construct made of two pipette tips nested inside each other without disruption of the power supply for the CE. The platinum content in the fractions was quantified offline with GF AAS, while the atomization took place at 2500 °C. For this purpose, the collected samples were diluted with double-distilled water without further mineralization of the sample. The hyphenation CE-GF AAS proved suitability for the detection of platinated DNA species (limit of detection: 0.78 ng) [32]. Apart from investigating the interaction with DNA, such an approach could be used to assess the binding to other macromolecules, which constitute potential targets of platinum drugs [37]. Moreover, this can be extended to other metal-containing drugs apart from representatives bearing platinum.

The two applications presented above involve an offline hyphenation of CE and AAS. Such an approach covers several manual operations but provides a certain degree of independence. Both methods, for separation and for detection, can be optimized without mutual influence. Moreover, the collected samples do not need to be quantified immediately after separation but later following a short period of storage. However, in the intervening time, contamination or degradation of the samples could occur. In some cases, additional mineralization of the collected fractions by dry or wet digestion using heat or strong acids, respectively, could become necessary. This would be time-consuming and cost-intensive and would contradict a green chemistry approach. In contrast, at least when employing ET AAS/GF AAS, the operation step of pyrolysis is aimed at removing organic matrix constituents, thus rendering mineralization unnecessary. Offline hyphenation is not completely automatable and therefore not best suited for real-time monitoring. The drawbacks of offline coupling can mostly be circumvented in the case of online hyphenation. Separation and quantification are made in one single sequence without interim sample storage, allowing for automatization and high sample throughput. However, both analytical techniques have to be compatible. In addition, an interface connecting both devices as well as an appropriate software for analysis of data are required. This is accompanied by higher acquisition expenses in the case of online hyphenation [38,39,40,41,42].

### 2.2. Examples of Online Hyphenation of CE-AAS

In pharmaceutical analysis, hyphenated analytical techniques gain in importance [43,44]. Nonetheless, the applications of CE-AAS are rare, but its potential in pharmaceutical analysis must be brought to light.

**Figure 2:**
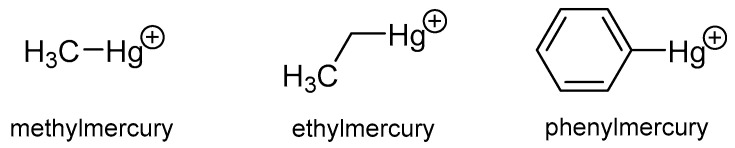
Chemical structures of organic mercury compounds whose binding to DNA was determined using online hyphenation of CE with ET AAS.

The study by Li and co-workers harnessed the online combination of CE with ET AAS to investigate the interaction of several mercury species with DNA. In particular, the binding of inorganic divalent mercury (Hg^2+^) as well as of organic derivatives of monovalent mercury (i.e., methylmercury, ethylmercury, and phenylmercury; see Figure 2) to herring sperm DNA was examined. Information on the thermodynamics and kinetics of the binding was deduced [45].

**Figure 3:**
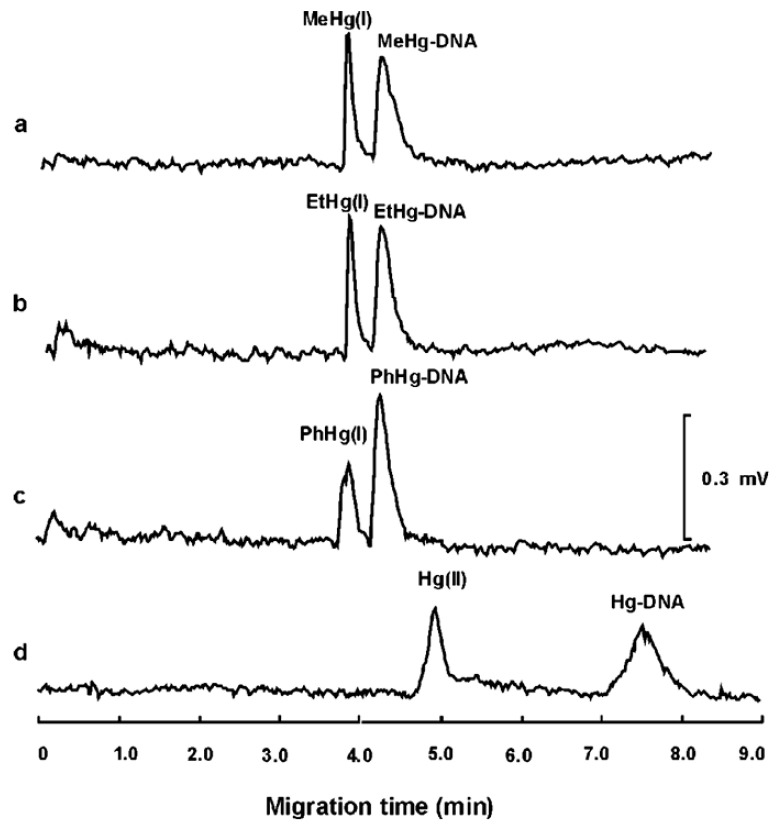
Electropherograms obtained by CE-ET AAS for the interactions of 2 µmol L^−1^ methylmercury (MeHg(I)), (**a**); ethylmercury (EtHg(I)), (**b**); phenylmercury (PhHg(I)), (**c**); and divalent mercury (Hg(II)), (**d**) with 4 µmol L^−1^ DNA (concentration of base pairs) after 12 h incubation. Copyright 2006 American Chemical Society and with kind permission [45].

Studying the behavior of mercury species on DNA is of interest as it bestows evidence to understand the mechanism of their toxicity. Especially methylmercury is known for its high neurotoxicity [46]. Heavy metals such as arsenic, cadmium, lead, and also mercury form stable complexes with biomolecules (e.g., DNA), resulting in changes of their three-dimensional molecule structure or even in denaturation, ultimately leading to loss of function [47]. If the formation of the complexes is accompanied by a change in electrophoretic mobility, CE is ideal to determine the binding. Employing CE, the mercury species were separated with good resolution (see Figure 3), and no derivatization was necessary for the detection with ET AAS.

For the AAS-based analysis, the time temperature program covered seven sequential steps, while each had the longest adjustable duration of 99 s. These steps followed each other and had a constant atomization temperature of 450 °C without gas flow. However, an interface connecting CE and AAS was required. The authors used a laboratory-made thermo-spray interface. They found that the interaction of the mercury species with the DNA was according to first-order kinetics for the mercury-containing compounds. Since an increase in the concentration of DNA did not result in a significant change in the kinetic rate constant, a zero-order kinetic profile was assumed for the DNA. A limit of detection of about 10^−8^ mol L^−1^ was found [45].

A similar approach of combining CE with ET AAS was followed to assess the stoichiometry of the interaction between the four mercury species mentioned above and human serum albumin (HSA) (limit of detection about 10^−8^ mol L^−1^). The investigation revealed a stoichiometry of mercury-containing compound to HSA of 6:1 for inorganic divalent mercury, 4:1 for methylmercury and ethylmercury, respectively, and 3:1 for phenylmercury [48]. Investigating the binding of a compound to HSA is crucial in pharmaceutical research. At a share of about 50%, serum albumin constitutes the most abundant protein in human plasma [49]. It serves as a primary carrier for drug molecules. Therefore, the pharmacokinetic profile of a drug and also its pharmacodynamic behavior in the organism are determined by the extent of binding to serum protein [50].

**Figure 4:**
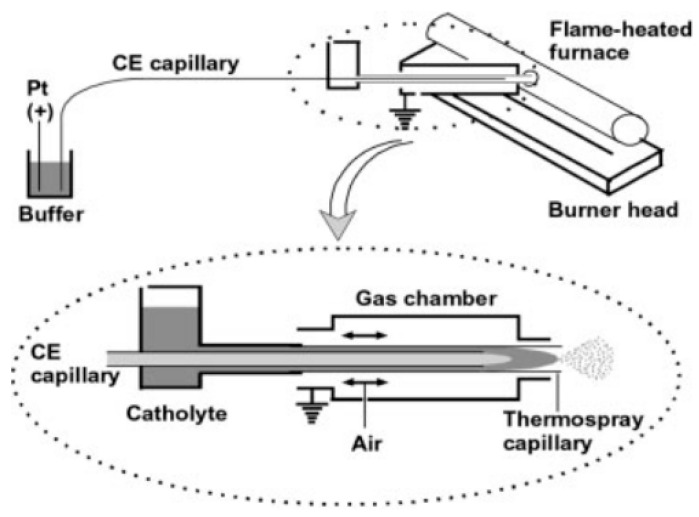
Schematic depiction of the hybrid technique hyphenating CE online with FHF AAS using a thermo-spray interface. Copyright 2005 Wiley-VCH Verlag GmbH & Co. KGaA, Weinheim and with kind permission [51].

In another study, the same authors specified traces of divalent mercury (dissolved in hydrochloric acid 1%, *v*/*v*) as well as methylmercury and phenylmercury (both dissolved in methanol) by CE hyphenated online with flame-heated furnace AAS (FHF AAS). A tube made of quartz served as the furnace. It was installed in the flame. The mercury-containing species separated by CE were vaporized straight into the tube. Therefore, a hole (diameter 2 mm) was centrally arranged in the tube. A custom-built interface was used for the transfer of the vapor (see Figure 4). It was composed of a catholyte cell and a gas chamber. The capillary of the CE ran through the catholyte compartment and opened into a stainless steel capillary. The latter served as a thermal spray device, protruding from the gas chamber and opening into the quartz tube. This ensured consistent vaporization and thorough transfer of the sample for the AAS detection. A detection limit of 53 µg L^−1^ (3.2 pg), 48 µg L^−1^ (2.9 pg), and 50 µg L^−1^ (3.0 pg) was reached for divalent mercury, methylmercury, and phenylmercury, respectively [51].

Due to the unique fact that mercury can be reduced to its atomic vapor already at room temperature without heating (so to speak in the cold) [52], the possibility of analyzing it with the cold vapor AAS (CV AAS) technique arises. Using such a vapor generation technique, it is feasible to improve the limit of detection [53]. A decrease by up to three orders of magnitude in comparison to conventional nebulization is suggested [21]. Limits of detection accounting for 0.027 µg mL^−1^ and 0.035 µg mL^−1^ of divalent mercury and methyl mercury, respectively, were obtained when specifying them in samples of dry goldfish. For this purpose, CE was coupled online to cold vapor generation with an AAS. The CE capillary passed through a hose made of Teflon. A solution of hydrochloric acid (HCl) was pumped through this hose, and a solution of sodium borohydride (NaBH_4_) through another Teflon hose attached below it. Both liquid streams, i.e., CE effluent/HCl and NaBH_4_, were combined in a wider hose surrounding the other two hoses. By mixing the two solutions, mercury vapor was generated. This vapor was directed using a tube made of hard glass straight into a furnace containing an electrothermally heatable quartz tube employing an argon flow. The liquids were collected in a basin. For further details regarding this sophisticated interface, reference to the original work is suggested [21].

**Figure 5:**
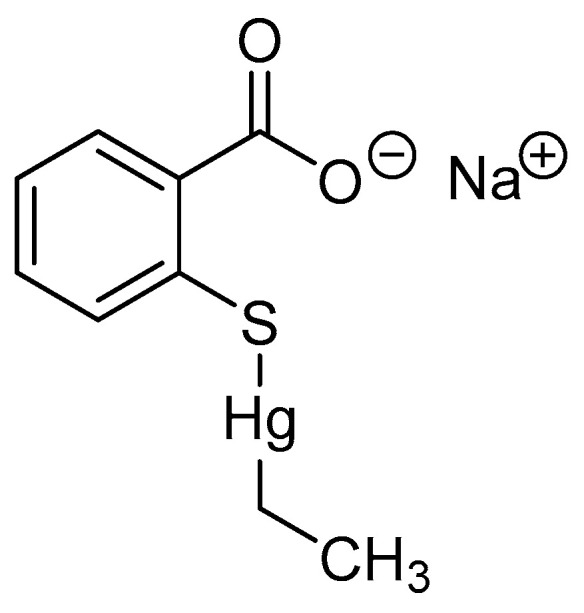
Chemical structure of thiomersal, which is used as a preservative in pharmaceutical preparations.

Given the high toxicity of mercury-containing drugs, there are hardly any of such compounds used for pharmaceutical purposes. However, the preservative thiomersal (also known as thimerosal; sodium ethylmercury thiosalicylate, see Figure 5) is nowadays still contained in few vaccines, although its use is restricted [54]. Thiomersal can generally be assessed in pharmaceutical products using CV AAS [55,56].

**Figure 6:**
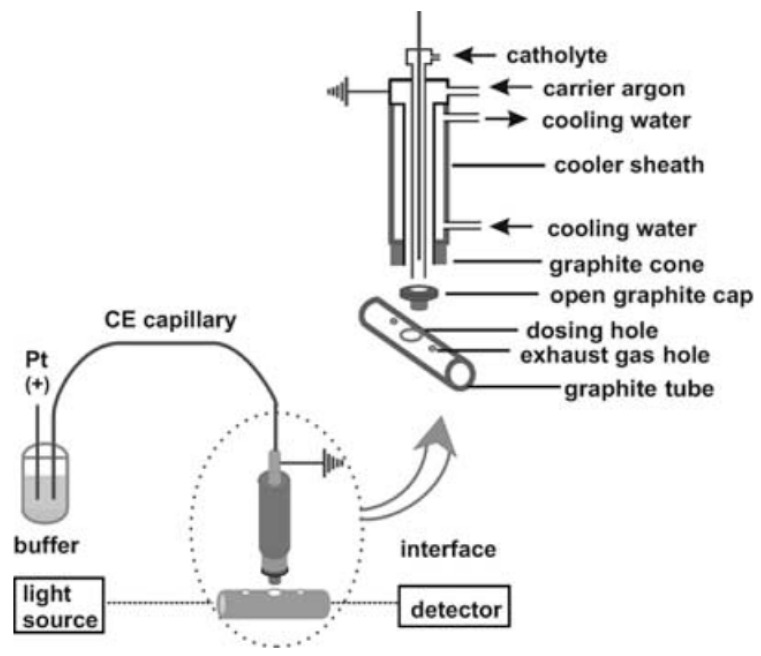
Schematic depiction of the hybrid technique hyphenating CE online with ET AAS using a thermo-spray interface. Copyright 2005 Wiley-VCH Verlag GmbH & Co. KGaA, Weinheim and with kind permission [57].

Similar to the study investigating the binding of mercury species to HSA, the research work to determine the interaction of mercury with DNA and divalent cadmium (Cd^2+^) with bovine serum albumin (BSA) was focused on CE hyphenated with ET AAS [57]. BSA plays an important role in pharmaceutical research as a cheaper surrogate of HSA [58]. As reported before, the graphite furnace was run in seven steps at constant temperature for the maximum time of 99 s. The temperature was 600 °C and 1000 °C for the atomization of mercury and cadmium, respectively. The separation and detection techniques were combined online using a homemade thermo-spray interface (see Figure 6).

For this purpose, the thermo-spray vaporizer entered into a hollow cap made of graphite (inner diameter 1.0 mm). The latter was inserted into an enlarged aperture of the dosing hole. To allow for escaping of exhaust gas, one hole on each side of the sample inlet whole were drilled into the tube [57]. The authors found that the limit of detection of mercury was independent of the mercury species (divalent mercury, methyl mercury, phenyl mercury; limit of detection: 14.8 µg L^−1^). In addition, employing the hyphenated technique, the effect of the pH value on the complexation of divalent cadmium by ethylenediaminetetraacetate (EDTA) was studied [57].

The stoichiometry of the interaction of divalent cadmium with DNA as well as its thermodynamics and kinetics was assessed by combining CE online with ET AAS. It was found that the stoichiometry of divalent cadmium interacting with DNA amounted to 1:5. Two binding sites on the DNA emerged and first-order kinetics revealed for cadmium binding to DNA. Strong affinity of divalent cadmium to DNA was demonstrated by binding constants of 10^6^ L mol^−1^ and 10^5^ L mol^−1^. Once again, a homemade thermo-spray interface was utilized. The goal was to obtain information on the mode of carcinogenicity and genotoxicity of the heavy metal. The temperature during the monitoring was 900 °C [59].

Another heavy metal whose binding to DNA was investigated harnessing CE hyphenated online with ET AAS is divalent lead (Pb^2+^). A homemade interface was employed. It is described in more detail in the literature [60,61]. Atomization took place at the temperature of 1700 °C. The detection limit accounted for 1.8 µmol L^−1^ of divalent lead. The study aimed to deduce the primary and the non-specific binding number as well as the respective binding constants. This is of interest as various metals might induce different changes of the DNA structure [23]. From a medicinal point of view, heavy metals such as cadmium and lead play a minor role. However, monitoring their thresholds is important to guarantee the quality of pharmaceutical preparations [62,63,64].

Apart from cadmium and lead, arsenic is also considered as a metal critical for health [65,66,67,68]. Inorganic arsenic species (i.e., trivalent and pentavalent arsenic) in sediment were quantified by coupling of CE online to hydride generation (HG) ET AAS. In this case as well, a suitable interface was designed. Two tubes (made of polytetrafluoroethylene) next to each other were used, while one was connected to a solution of sodium borohydride (NaBH_4_). The other tube had a capillary inside it. A solution of hydrochloric acid (HCl) served to carry the CE effluent or to support hydride generation. This solution flowed between the tube and the CE capillary. A ceramic tube was used to establish the connection to the graphite tube of the graphite furnace. A defined time-temperature program was followed to heat the furnace, with atomization taking place at 1700 °C. The elaborated online CE-AAS method applying a self-designed interface proved to be competitive for separating and quantifying (limit of detection: <160 ng g^−1^) As^3+^ and As^5+^ [20].

**Figure 7:**
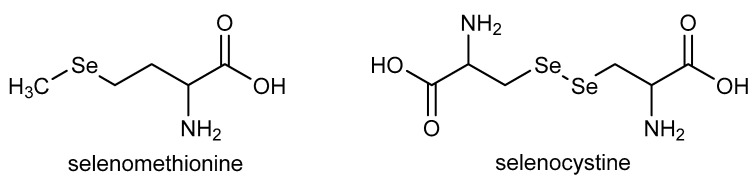
Chemical structures of organic selenium compounds analyzed in rhizomes of *Zingiber officinale* using online hyphenation of CE with ET AAS.

From a pharmaceutical perspective, arsenic comes into play, for example, in the context of analyzing raw materials of medicinal plants [14]. The online combination of CE with ET AAS was applied to specify selenium species in rhizomes of a pharmaceutically used plant, i.e., ginger (*Zingiber officinale*) before [60]. Ginger is known for its valuable pharmacological effects, e.g., anti-inflammatory activity [69,70] and antioxidant properties [71,72]. In their study, Deng and coworkers separated the inorganic selenium species selenite (SeO_3_^2−^) and selenate (SeO_4_^2−^) as well as organic selenomethionine and selenocystine (see Figure 7), reaching detection limits of 0.89 ng mL^−1^, 0.97 ng mL^−1^, 1.7 ng mL^−1^, and 2.2 ng mL^−1^, respectively. The atomization was performed at a temperature of 1900 °C. A sophisticated interface was crafted to connect the CE with the AAS. Ultimately, the sample was introduced into the graphite tube using a ceramic tube that laterally protruded into the graphite tube. The actual centered hole of the graphite tube was covered with a T-shaped plug made of graphite [60].

The quantification of different selenium-containing compounds in ginger is of general interest, since they can be either harmful or essential, dependent on the particular selenium compound and the concentration [73]. Organic selenium compounds are less toxic than inorganic ones [74]. This illustrates the importance of separating the different chemical forms using CE. For medicinal plants, the range between essential and toxic selenium concentrations is also narrow [75,76].

Another study employing CE coupled online with ET AAS focused on the analysis of selenium species in wastewater and juice from fermented bean curd. The limits of detection accounted for 0.18 ng mL^−1^, 0.17 ng mL^−1^, 0.54 ng mL^−1^, and 0.49 ng mL^−1^ for selenite, selenate, selenomethionine, and selenocystine, respectively. Such competent detection limits were reached following an extraction using silica-coated magnetic nanoparticles modified with 5-sulfosalicylic acid. The CE effluent was introduced onto the graphite tube through its side opening. The upper hole of the tube was sealed with a cone plug made of graphite, thus diminishing losses and hence raising the sensitivity [77]. The spray interface used for this purpose is represented by a concentric nebulizer as described for analyzing calcium in human plasma by CE connected with inductively coupled plasma (ICP) optical emission spectrometry (OES) [61]. The electrothermal atomization of selenium was achieved at 1900 °C, while the time-temperature program of heating the graphite furnace included operation steps of distinctly lower temperatures (i.e., 300 °C for ashing) [77].

During the latter stage, pyrolysis of concomitant matrix compounds is generally performed. This process takes place prior to the actual measurement (i.e., during the atomization step) and is necessary when a new sample is introduced into the graphite furnace. It can be concluded that AAS has only limited suitability for continuous online detection of the CE effluent. Due to the quite long duration of sustained use at temperatures as high as for atomization, the lifetime of the graphite devices (e.g., tube, furnace) is drastically reduced [78]. Since graphite furnaces are not intended for continuous run, the design of the interface is crucial to allow for the combined use of CE and ET AAS [57,60]. This is achieved by introducing the sample into the spectrometer with a thermo-spray interface [79]. However, this drawback does not apply in the case of offline hyphenation.

### 2.3. Discussion

In summary, it can be said that due to inherent shortcomings of traditional AAS instruments, an appropriate interface is required for the online connection to CE [19]. Nevertheless, the usage of interfaces such as described above has several advantages. There is no need for an additional cooling system between the two instruments. As the effluent from the CE representing the samples for ET AAS stays longer in the graphite furnace, the efficiency of atomization is enhanced. Moreover, continuous detecting generally becomes feasible compared to pulse detection only [77].

In most of the presented cases (see Table 1), hyphenation was made to ET AAS and GF AAS, respectively, owing to its wide range of application and its competitiveness concerning low limits of detection and high sensitivity [80]. Compared to F AAS, where detection limits in the parts per million (ppm) range are obtained, ET AAS/GF AAS is about 10^3^ times more sensitive. The detection limits of the latter are within the parts per billion (ppb) range [52]. Therefore, electrothermal atomization in a graphite tube is generally more highly recommended than atomization using a burner flame. A further aspect in favor of ET AAS/GF AAS is the lower sample volume compared to F AAS or HG AAS.

Another approach for detection would be ICP-based methods. However, higher expenses for the instruments themselves as well as greater operational costs compared to AAS [81] restrict the usage for routine analysis [82]. Moreover, well-trained staff are required for the operation, thus potentially limiting its wide application [21,23].

In contrast, the advantages of CE-AAS encompass simplicity of implementation and low costs for acquisition and operating [21,23]. In addition, the hyphenation impresses with both efficient resolution and high sensitivity. Only small volumes of samples and reagents are consumed [19]. The analysis times are quick. No lengthy dialysis sessions are required and potential interferences caused by other metals can be prevented following the separation [23].

A CE-based separation can be performed under physiological buffer conditions, required to study biomolecules [45]. The buffer constituents or other ingredients, such as cellular components, are easily removed during the ashing step of ET AAS, represented by a suitably increased pyrolysis temperature [83]. However, this context also carries the risk that salts contained in the running buffer of CE interfere with the AAS procedure. Common buffers in CE are phosphate buffers (for example applied in the presented studies [20,57,60,77]) and boric acid and borate buffers (used in the studies [21,45,48,51]), the usage of tris(hydroxymethyl)aminomethane (TRIS) or TRIS-HCl (e.g., in the investigations [23,45,48,59]), ammonium acetate (e.g., applied in study [59]) as a background electrolyte (BGE), or sodium dodecyl sulfate (SDS) adduced as an additive [84,85].

Phosphates are known to form refractory, thermally stable compounds (e.g., pyrophosphates) with several metal cations such as calcium or magnesium. Heat-resistant compounds do not atomize properly and thus cause suppression of the absorption signal. Another issue with phosphate-based buffers is represented by background interferences due to PO molecular absorption or Zeeman effect overcorrection, which occurred in the analysis of lead [86,87].

Chlorides, for example present in TRIS-HCl, were found to reduce the absorption signal when analyzing cadmium. This was related to the formation of cadmium chloride. It was suggested that this salt does not dissociate quantitatively, hence diminishing the number of free cadmium atoms [88].

While sulfate-based buffers are used less frequently in CE, SDS is often applied in the analysis of proteins [89]. Sulfates are discussed to form palladium sulfate in the presence of palladium [90]. The latter is a universally used modifier in AAS [91]. It is assumed that upon generation of palladium sulfate analytes such as selenium can no longer be stabilized as efficiently, resulting in loss of absorption signal [90].

These examples demonstrate that, dependent on the analyte to be assessed with AAS, the choice of the CE buffer must be carefully considered. On the contrary, ingredients of the CE buffer could also serve as a modifier and therefore support atomization. However, this is a matter of the particular metal. The selection of a CE buffer suitable also for the AAS analysis therefore depends on the metal to be analyzed.

Another advantage is given from a green chemistry perspective, because the use of high amounts of hazardous organic solvents does not arise in the case of CE-AAS [22,51].

The CE-AAS combination technique offers a wide range of applications (see Table 1), also for investigations relevant to pharmaceutical sciences. These include, for example, the separation and quantification of different metal species in medicinal plants as well as determining the binding behavior of metals in general or metal-containing drugs to proteins such as HSA or to the DNA. Future studies could also investigate the extent of binding to the iron-specific carrier protein transferrin. It was found that iron-containing compounds with anticancer or antibacterial activity exhibit a high binding to transferrin. This suggests facilitating the uptake into cancer or bacterial cells [92,93,94]. In general, iron complexes demonstrate great promise as antimicrobial drugs [95]. In this context, ferroptosis induced by reactive oxygen species following the Fenton reaction is discussed as potential mechanism of action. However, only ferrous (divalent) iron can drive this reaction, but not ferric (trivalent) iron [96]. Therefore, differentiation of these iron species and separation using CE is of importance. Generally, it should be noted that other metal-containing compounds may also bind to transferrin, e.g., the clinically widely used anticancer drug cisplatin [97].

Oxidative damage plays a role also in Wilson’s disease. It is characterized by an improper transport of copper, resulting in accumulation of free copper for example in the liver and causing oxidative organ damage [98]. Normally, copper is bound to the glycoprotein ceruloplasmin and non-bound copper could be used as a biomarker for the diagnosis and the management of Wilson’s disease [99]. This could be investigated with CE-AAS, too. Similar studies may also focus on metallothioneins [100].

The hyphenation of CE with AAS could be exploited to monitor superparamagnetic iron oxide nanoparticles considered for use in diagnosing and treating cancer [101] or, more generally, other nanoparticles as well.

All these kinds of investigations provide information on composition, (im)purity, toxicity, circular distribution, and potentially on the mode of action. In the latter case, metalloproteins often play an important role [102,103] or they are included in medicinal plants. Such phytopharmaceuticals could be interested based on the biological effects of their extracts [104]. As different qualities of water are essential for the pharmaceutical industry [105], it could be considered to apply also CE-AAS in the context of water analysis. Water analysis employing CE is already common practice [6], and AAS is also used [106]. By combining both techniques, as described above, the limitations of each individual technique can be overcome.

The question arises as to which approach, offline or online, can be recommended for combining CE and AAS for pharmaceutical analysis. Their respective advantages and disadvantages were mentioned above. Given the fact that interfaces are not commercially available thus far, offline hyphenation seems to be advisable. However, in some cases offline hyphenation, which requires for collection of the CE effluents, is not possible. This applies mainly to samples that are sensitive to the storage, even if only briefly. In addition, offline connection of CE to AAS is not possible with HG AAS or CV AAS. The hydrides formed in terms of these sub-techniques are highly volatile and therefore susceptible to loss during offline treatment. It is questionable whether the analytes accessible with HG AAS (antimony, arsenic, bismuth, selenium, tellurium, tin) and CV AAS (mercury) implicitly need to be detected with these particular sub-techniques. All these metals can also be measured using ET AAS/GF AAS. The latter allows for an offline approach. Its shortcomings were mentioned before and can be overcome in an online procedure. That would be even better if the design of interfaces necessary for online combination was further refined, with respect to a potential commercial acquisition. If this succeeds and if researchers become aware of the benefits of combining CE with AAS, then CE-AAS will become a highly valuable tool, also for investigations in the pharmaceutical sciences.

## 3. Conclusions

CE and AAS represent analytical techniques of high performance and are thus each widely used in pharmaceutical industry and drug-related research. The hyphenation of both these techniques, however, is rarely done, although they can complement each other. In the current perspective, examples are presented of cases in which the hybrid technique CE-AAS has already been applied so far. The objective was to establish a connection to pharmaceutical applications and thus to demonstrate the potential impact of CE-AAS on pharmaceutical analysis. The examples of usage cover investigations to study the interaction of metals with DNA or the binding to HSA or BSA, as well as the analysis of aqueous samples or quantifying the metal content in plants with medicinal properties. In general, the separation based on CE and the limits of detection during detection employing AAS yielded competitive results, thus suggesting suitability of the hybrid technique. For the selection of the CE buffer, its potential interferences in the AAS-based measurement must also be taken into account. Except for offline combination, the online hyphenation of CE with AAS still faces the challenge of an appropriate interface. The studies described thus far used homemade interfaces, whose sophistication might not yet be so easy to convert to routine analysis. It remains to be hoped that the availability of interfaces, including ones available for commercial purchase, will improve in the future. This will allow even better usage of the CE-AAS hybrid technique, for pharmaceutical use and beyond.

## Figures and Tables

**Table 1:** Overview of representative studies in which the hyphenated technique CE-AAS was used.

Metal	Hyphenation	AAS Sub-Technique	Investigation/Sample	Reference
Cr	offline	ET AAS	aqueous samples	[24]
Pt	offline	GF AAS	binding of anticancer complex to DNA	[32]
Hg	online	ET AAS	interaction with DNA	[45]
Hg	online	ET AAS	binding to HSA	[48]
Hg	online	FHF AAS	hydrochloric and methanolic samples	[51]
Hg	online	CV AAS	dry goldfish muscle	[21]
Hg, Cd	online	ET AAS	binding to BSA, EDTA	[57]
Cd	online	ET AAS	interaction with DNA	[59]
Pb	online	ET AAS	binding to DNA	[23]
As	online	HG ET AAS	sediment	[20]
Se	online	ET AAS	rhizomes of ginger (*Zingiber officinale*)	[60]
Se	online	ET AAS	wastewater and juice from fermented bean curd	[77]

## Data Availability

Data sharing is not applicable.

## Abbreviations

The following abbreviations are used in this manuscript:

AAS	Atomic absorption spectrometry
BGE	Background electrolyte
BSA	Bovine serum albumin
CE	Capillary electrophoresis
CV AAS	Cold vapor atomic absorption spectrometry
DNA	Deoxyribonucleic acid
EDTA	Ethylenediaminetetraacetate
ET AAS	Electrothermal atomic absorption spectrometry
F AAS	Flame atomic absorption spectrometry
FHF AAS	Flame-heated furnace atomic absorption spectrometry
GF AAS	Graphite furnace atomic absorption spectrometry
HG AAS	Hydride generation atomic absorption spectrometry
HSA	Human serum albumin
ICP	Inductively coupled plasma
OES	Optical emission spectrometry
ppb	Parts per billion
ppm	Parts per million
SDS	Sodium dodecyl sulfate
TRIS	Tris(hydroxymethyl)aminomethane
